# Pulmonary diesel particulate increases susceptibility to myocardial ischemia/reperfusion injury via activation of sensory TRPV1 and β1 adrenoreceptors

**DOI:** 10.1186/1743-8977-11-12

**Published:** 2014-02-25

**Authors:** Sarah Robertson, Ashleigh L Thomson, Rod Carter, Holly R Stott, Catherine A Shaw, Patrick W F Hadoke, David E Newby, Mark R Miller, Gillian A Gray

**Affiliations:** 1 BHF/ University Centre for Cardiovascular Science, Queens Medical Research Institute, University of Edinburgh, Edinburgh, Scotland, UK; 2 Toxicology Department, Public Health England, Harwell, Oxfordshire, UK

**Keywords:** (3-10) air pollution, Ischemia/reperfusion injury, Sympathetic nervous system, Vanilloid receptor

## Abstract

**Background:**

Clinical studies have now confirmed the link between short-term exposure to elevated levels of air pollution and increased cardiovascular mortality, but the mechanisms are complex and not completely elucidated. The present study was designed to investigate the hypothesis that activation of pulmonary sensory receptors and the sympathetic nervous system underlies the influence of pulmonary exposure to diesel exhaust particulate on blood pressure, and on the myocardial response to ischemia and reperfusion.

**Methods & Results:**

6 h after intratracheal instillation of diesel exhaust particulate (0.5 mg), myocardial ischemia and reperfusion was performed in anesthetised rats. Blood pressure, duration of ventricular arrhythmia, arrhythmia-associated death, tissue edema and reperfusion injury were all increased by diesel exhaust particulate exposure. Reperfusion injury was also increased in buffer perfused hearts isolated from rats instilled *in vivo*, excluding an effect dependent on continuous neurohumoral activation or systemic inflammatory mediators. Myocardial oxidant radical production, tissue apoptosis and necrosis were increased prior to ischemia, in the absence of recruited inflammatory cells. Intratracheal application of an antagonist of the vanilloid receptor TRPV1 (AMG 9810, 30 mg/kg) prevented enhancement of systolic blood pressure and arrhythmia *in vivo*, as well as basal and reperfusion-induced myocardial injury *ex vivo*. Systemic β_1_ adrenoreceptor antagonism with metoprolol (10 mg/kg) also blocked enhancement of myocardial oxidative stress and reperfusion injury.

**Conclusions:**

Pulmonary diesel exhaust particulate increases blood pressure and has a profound adverse effect on the myocardium, resulting in tissue damage, but also increases vulnerability to ischemia-associated arrhythmia and reperfusion injury. These effects are mediated through activation of pulmonary TRPV1, the sympathetic nervous system and locally generated oxidative stress.

## Background and objectives

Exposure to air pollution is associated with increased cardiovascular mortality and morbidity [1]. These associations are strongest for the particulate matter (PM) in air pollution, and exist even when exposure is only acute (reviewed in [2]). Ultrafine particles (particles with a diameter of less than 100 nanometers, UFP or PM0.1) are of specific concern because their small size engenders them with a large reactive surface area and allows them to penetrate deep into the respiratory tract [3]. Exhaust from diesel engines is especially rich in ultrafine particles and therefore may contribute greatly to the health effects of PM in urban environments [4].

The proposed link between air pollution and increased incidence of fatal and non-fatal myocardial infarction (MI) has recently been confirmed in a systematic review and meta-analysis [5]. This observation corresponds with growing clinical and experimental evidence for detrimental effects of diesel exhaust and other PM on blood pressure, coronary vascular function, atherosclerotic plaque development and stability, and on thrombosis and clot resolution, all factors that will increase the likelihood of MI [6]. However, epidemiological evidence suggests that there is more to increased cardiovascular death than enhanced risk of MI. In the Women’s Health Initiative [7] and the Nurses’ Health Study [8], the impact of PM exposure on cardiovascular mortality was much larger than for incidence of MI events. The evidence suggests a biological mechanism for PM that also involves effects on the myocardium itself. Such a mechanism may account for the increased incidence of ventricular arrhythmias reported in patients with implantable defibrillators following exposure to pollution or PM [9]. Experimental studies support a damaging effect of PM on the myocardium, as exposure *in vivo* causes impairment in cardiac function and arrhythmia [10-12]. In mice, both concentrated ambient particles [13] and particulate matter [14], increase susceptibility to ischemia/reperfusion (I/R) injury.

The mechanism by which pulmonary exposure to particulate matter is associated with myocardial injury and susceptibility to I/R is currently unclear. Systemic inflammation, occurring secondary to pulmonary inflammation, is a potential candidate for transmission of PM effects from the lung to the heart. However, evidence supporting the occurrence of systemic inflammation varies in the literature and is more compelling for chronic, rather then acute PM exposure [1,15]. Alternatively, exposure of the lung to PM might influence distant organs via neurohumoral activation [12]. Exposure to PM is associated with elevation of blood pressure [16,17] and there is evidence that this is mediated by activation of the sympathetic nervous system [18]. Autonomic outflow from the central nervous system (CNS) can be adjusted by sensory feedback from the lung [19], and several studies have demonstrated the ability of PM to activate these sensory pathways [10,11,20,21].

The hypothesis investigated in the present study is that activation of sensory transient receptor vanilloid receptors and of adrenergic receptors link pulmonary exposure to diesel exhaust particulate (DEP) with hemodynamic perturbation, myocardial injury and the response to myocardial ischemia and reperfusion.

## Results

### Pulmonary exposure to DEP increases blood pressure, ischemic arrhythmia, infarct size and mortality

**Figure 1:**
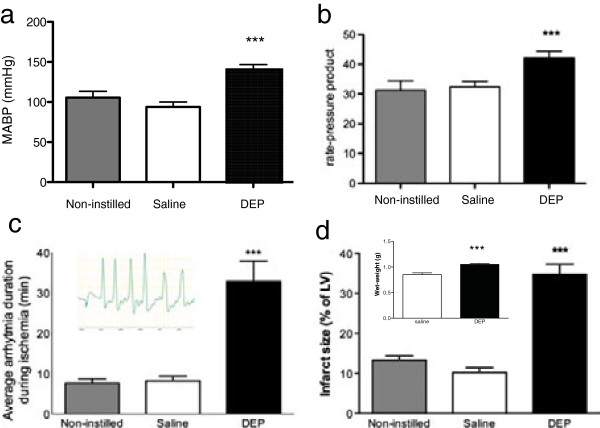
**Pulmonary exposure to DEP increases basal blood pressure, oxygen demand, ischemic arrhythmia and reperfusion injury *in vivo*.** Mean arterial blood pressure (MABP, **a**), rate pressure product (RPP, **b**) and the duration of ischemic arrhythmia (**c**, with inset showing typical arrhythmia) were measured in anesthetised rats 6 h after pulmonary instillation of diesel exhaust particulate (DEP, 0.5 mg, filled black columns) or 0.9% saline (saline, 0.5 ml, open columns) and in control rats that received no instillation (non-instilled, filled grey column). Hearts from DEP instilled rats had increased infarct size **(d)**, expressed as a percentage of left ventricular (LV) mass) and were heavier at the end of reperfusion (**d**, inset). Results are expressed as mean ± SEM (n = 6 per group) ***P < 0.001 saline versus DEP; one-way ANOVA followed by Bonferroni post-hoc test.

Systolic, diastolic and mean arterial pressures, as well as rate-pressure product (RPP), were all elevated in rats 6 h after DEP compared to saline or no instillation, but there was no significant change in heart rate. (Figure 1a and b, Additional file 1: Table S1). No arrhythmias were recorded during the baseline period before induction of I/R in any of the experimental groups.

Induction of ischemia by coronary artery ligation resulted in a typical transient drop in systolic blood pressure that returned to pre-ischemic levels within 10-15 min, this was accompanied by an increase in RPP (Additional file 1: Table S2). Arrhythmias were detected within 5 min of ischemia induction and typically lasted for 8 ± 1 min (Figure 1c). Prior instillation of DEP increased total arrhythmia duration to 33 ± 5 min (P < 0.01), while saline instillation had no effect (Figure 1c). Arrhythmias were accompanied by death within 15 min of the onset of ischemia in 60% of the rats that had received DEP. Sudden cardiac death did not occur in either non-instilled or saline-instilled animals.

Infarct size was assessed after 2 h of reperfusion. While Evans Blue exclusion could be used to delineate area at risk (AAR) in saline-instilled animals, this was not the case in animals that had received DEP. In these animals only, Evans Blue consistently leaked into the AAR at the end of reperfusion. An accompanying increase in heart weight (Figure 1d, inset) in DEP instilled animals following ischemia and reperfusion suggests that enhanced capillary permeability is likely to underlie leakage of Evans Blue into the ischemic zone. In all subsequent *in vivo* studies Evans Blue was not therefore administered and infarct size was calculated as % left ventricular (LV) mass. Infarct size in non-instilled animals averaged 13 ± 1% of total LV mass (Figure 1d), and this was not changed by saline instillation. In contrast, infarct size in DEP-instilled rats was increased threefold to 35 ± 2% of LV mass (P < 0.001).

### Neutrophil priming and recruitment do not account for effects of pulmonary DEP on the heart

White blood cell (WBC) concentration was increased in blood collected 6 h after DEP instillation (P < 0.05, See Additional file 1: Methods and Additional file 1: Figure S1a), whereas red blood cells (RBC) and platelet concentrations did not differ between groups (data not shown). Elevation of WBC was not associated with any change in the plasma concentration of the neutrophil chemoattractant CXCL8 (See Additional file 1: “Methods” and Additional file 1: Figure S1b). Flow cytometric analysis of whole blood, to assess whether systemic neutrophil priming contributed to DEP-enhanced myocardial injury, did not reveal any difference in either basal or fMLP-induced neutrophil activation (CD11b expression) between groups (Additional file 1: Figure S1c). Histological evaluation of sections from hearts collected 6 h after DEP instillation showed no evidence of inflammatory cell recruitment to the heart prior to induction of ischemia (data not shown).

### Susceptibility to ischemic injury is retained when the heart is perfused ex vivo

To determine the dependence of increased I/R injury on concurrent inflammatory cell recruitment or neurohumoral influences *in vivo*, hearts were isolated from rats 6 h after DEP or saline instillation, or from non-instilled rats, and buffer-perfused *ex vivo* in Langendorff mode.

**Figure 2:**
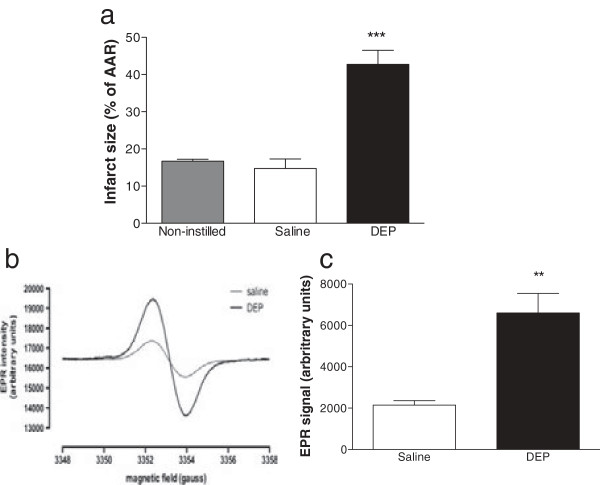
**Infarct size and oxidant stress generation was increased in hearts isolated from DEP instilled rats and perfused *ex vivo*.** Myocardial infarct size (**a**, expressed as percentage of area at risk; AAR) and **(b, c)** oxygen free radicals (via quantification of CP˙ spin adducts by electron paramagnetic resonance; EPR) in coronary perfusate of hearts from non-instilled rats (non-instilled, grey column) and hearts isolated 6 h after instillation of saline (saline, 0.5 ml, open columns) or DEP (0.5 mg, black column). Results are expressed as mean ± SEM (n = 4-6) **P < 0.01, ***P < 0.001 saline versus DEP; one-way ANOVA followed by Bonferroni post-hoc test.

Neither baseline coronary perfusion pressure (CPP), nor AAR after induction of ischemia, was different between groups (Additional file 1: Table S3). Leakage of Evans Blue into the AAR did not occur in hearts from DEP instilled animals, in contrast to *in vivo* studies, allowing reperfusion injury to be calculated at % AAR. In agreement with *in vivo* observations, infarct size following ischemia and reperfusion *ex vivo* was increased in hearts from DEP-instilled rats relative to hearts isolated from saline-instilled or non-instilled rats (P < 0.001; Figure 2a).

**Figure 3:**
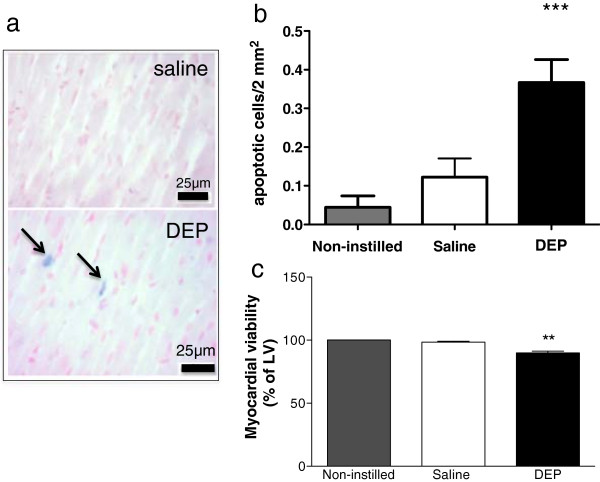
**Intratracheal DEP instillation increased apoptotic cell death and reduced cardiomyocyte viability. (a)** Representative images of left ventricle from rats 6 h after instillation of DEP (0.5 mg, lower panel) or saline (0.5 ml, upper panel) showing blue staining of TUNEL-positive apoptotic cells. DEP instillations (black columns, **b**) increased the number of apoptotic cells in the heart and **(c)** decreased the area of viable myocardium (% of left ventricle (LV)), compared to hearts from non-instilled (grey columns) and saline instilled (open columns) rats. Results are expressed as mean ± SEM (n = 3-4) **P < 0.01, **P < 0.001, saline versus DEP; one-way ANOVA followed by Bonferroni post-hoc test.

Oxidant stress, determined by electron paramagnetic resonance (EPR) of the heart perfusate before induction of ischemia and reperfusion, was higher in hearts from DEP-instilled rats (P < 0.01; Figure 2b & c). All hearts showed a small burst of oxidant stress on reperfusion but this did not differ between treatment groups (data not shown). In separate hearts, fixed 6 h after DEP instillation and prior to I/R, detection of TUNEL-labelled apoptotic cells was increased relative to hearts from non-instilled or saline instilled hearts (P < 0.01; Figure 3a & b), while assessment of tetrazolium chloride staining showed a corresponding loss in myocardial cardiac cell viability (P < 0.01, Figure 3c).

### Systemic β_1_ adrenoreceptor blockade prevents DEP-induced cardiac injury and promotion of reperfusion injury ex vivo

**Figure 4:**
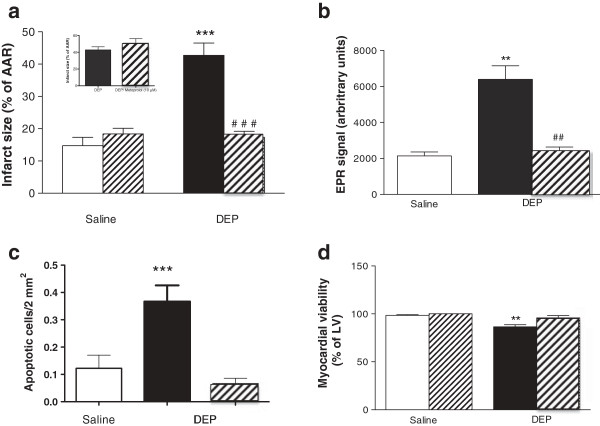
**β**_**1 **_**adrenoceptor blockade *in vivo *prevented the effects of intratracheal DEP on infarct size, myocardial oxidant stress, apoptosis, cell viability *ex vivo*.** Infarct size was reduced in hearts isolated from DEP instilled (filled columns) compared to controls (open columns) rats when metoprolol was co-administered *in vivo* (10 mg/kg, i.p., hatched columns **a**), but not when metoprolol was present only in the perfusate *ex vivo* (10 μM, hatched column, **(a)** inset panel). The DEP-induced changes in oxygen-derived free radicals in the coronary perfusate, (electron paramagnetic resonance; EPR, **b**), number of apoptotic cells (TUNEL staining, **c**), and loss of cardiomyocyte viability (TTC staining, **d**) in the left ventricle (LV) were prevented when metoprolol (10 mg/kg, i.p., hatched columns) was administered *in vivo* at the time of instillation. Results are expressed as mean ± SEM (n = 6), **P < 0.01, ***P < 0.001 versus saline; ^##^P < 0.01, ^###^P < 0.001 versus DEP without metoprolol; two-way ANOVA followed by Bonferroni post-hoc test.

The role of the sympathetic nervous system in mediating the effects of DEP was investigated by administration of the β_1_ adrenoreceptor selective antagonist, metoprolol (10 mg/kg i.p.), at the time of DEP instillation *in vivo*. In hearts isolated and buffer-perfused 6 h later, neither the baseline perfusion pressure nor the AAR after ischemia was influenced by prior *in vivo* β_1_ adrenoreceptor blockade (Additional file 1: Table S4). However, an influence of DEP on *ex vivo* reperfusion injury was absent in rats that had received metoprolol at the time of DEP instillation *in vivo* (Figure 4a). Hearts were not protected when metoprolol was added only to the perfusate *ex vivo* (Figure 4a, inset), confirming that protection occurs as a result of prevention of β_1_ adrenoreceptor activation *in vivo. In vivo* treatment with metoprolol was also effective in reducing DEP-associated oxygen free radical generation (P < 0.01, Figure 4b), apoptotic cell death (Figure 4c), and the corresponding reduction in cardiomyocyte viability prior to I/R (Figure 4d).

### Pulmonary administration of a TRPV1 channel antagonist in vivo prevents DEP-induced enhancement of myocardial injury ex vivo

**Figure 5:**
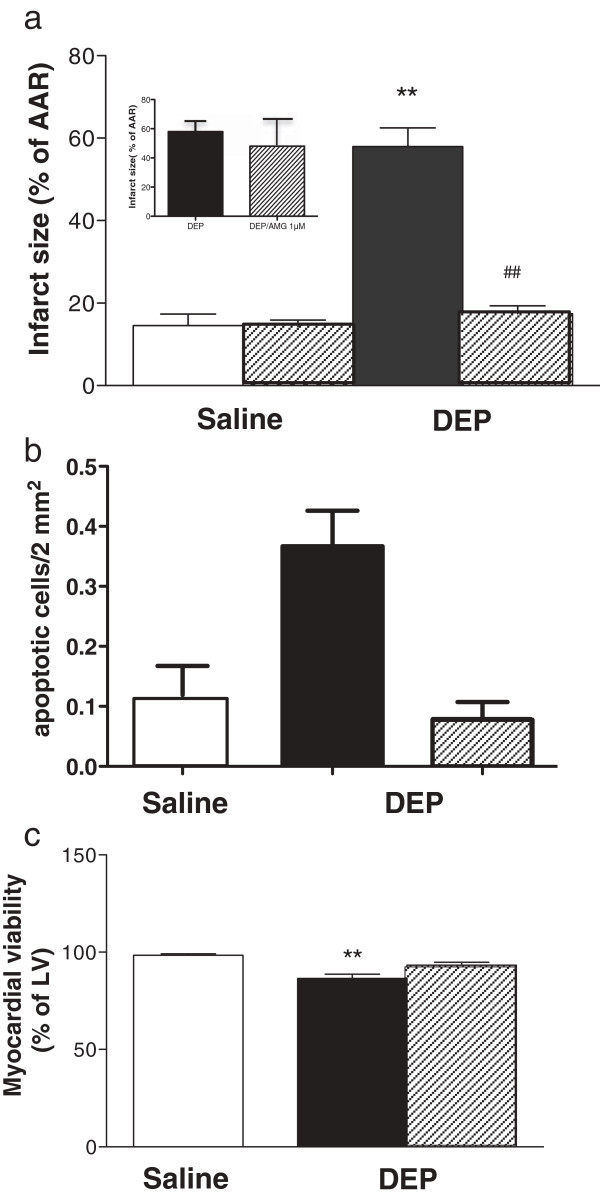
**Pulmonary TRPV1 blockade *in vivo *prevented the effects of intratracheal DEP on infarct size, myocardial apoptosis and cell viability *ex vivo*.** Co-instillation of the TRPV1 antagonist AMG 9810 (hatched columns, 30 mg/kg) with DEP *in vivo* prevented the increase in myocardial infarct size (expressed as a percentage of area at risk; AAR) induced by DEP (0.5 mg, black column, **a**). AMG9810 had no effect on infarct size when present only in the perfusate *ex vivo* (1 μM, hatched column, **(a)** inset panel). When co-administered *in vivo* AMG 9810 also prevented the greater levels of apoptosis (TUNEL staining, **b**) and loss of cell viability (TTC staining, **c**) associated with pulmonary DEP exposure. Results are expressed as mean ± SEM (n = 3-5), **P < 0.01 versus saline; ^##^P < 0.01 versus DEP without AMG 9810; two-way ANOVA followed by Bonferroni post-hoc test.

The role of pulmonary TRPV1 receptors in mediating the influence of DEP on the myocardium was investigated by co-instillation *in vivo* of the TRPV1 antagonist AMG 9810 (30 mg/kg) with DEP into the lung. Enhancement of *ex vivo* reperfusion injury associated with DEP in buffer-perfused hearts was prevented by AMG 9810 co-instillation *in vivo* (Figure 5a). Treatment had no influence on either baseline CPP or AAR (Additional file 1: Table S4). Furthermore, AMG 9810 had no influence on injury in hearts from DEP-instilled rats when given only *ex vivo* in the Langendorff perfusate, confirming that the effects of this drug intervention were mediated prevention of TRPV1 activation *in vivo* (Figure 5a inset). Increased apoptosis (Figure 5b) and reduced cardiac viability (Figure 5c) associated with hearts from DEP-instilled rats were also prevented by AMG 9810 treatment *in vivo*.

### Pulmonary TRPV1 channel blockade prevents the DEP-induced enhancement of blood pressure and arrhythmia in vivo

To investigate whether TRPV1 blockade might also be a means of preventing the *in vivo* effects of DEP, AMG 9810 (30 mg/kg) was co-instilled with DEP into the lung 6 h prior to anesthesia and induction of I/R. AMG 9810 co-treatment prevented the increase in blood pressure associated with DEP administration (SBP: 131 ± 8 mmHg, n = 4 after DEP compared to 101 ± 9 mmHg, n = 4 after saline instillation, P < 0.05, and 112 ± 10 mmHg, n = 5 with DEP after AMG9810). Co-administration of AMG also tended to reduce the duration of ischemic arrhythmias in DEP-instilled animals, from 17 ± 12 min to 8 ± 1 min, comparable with duration in saline instilled rats (6 ± 2 mmHg). AMG 9810 itself had no significant effect on blood pressure (110 ± 7 mmHg, n = 4) or ischemic arrhythmia duration (8 ± 1 min) in saline-instilled animals. These results suggest that TRPV1 activation underlies the increases in blood pressure and arrythmogenesis associated with DEP instillation.

## Discussion

The link between environmental air pollution and cardiovascular mortality is now clear, but the mechanisms are complex, varying, and not completely elucidated [1,22]. The current study demonstrates that, within a few hours of pulmonary exposure to DEP, there is a significant increase in blood pressure, damage to the myocardium and an increase in sensitivity to I/R induced myocardial injury and arrhythmia. Hearts isolated from animals exposed to DEP *in vivo* produce more oxidant stress and retain increased vulnerability to subsequent injury induced *ex vivo*. Outcomes from pharmacological intervention studies support a role for pulmonary sensory TRPV1 receptors and also β1 adrenoreceptors, in mediating these effects.

Elevation of blood pressure has been widely reported in clinical and experimental studies involving both acute and chronic exposure to environmental pollutants [22,23]. Diesel exhaust, and other sources of urban air pollution, represent complex mixes of gases, semi-volatile liquids and particulates. Multiple epidemiological studies have demonstrated that the associations between air pollution and cardiovascular disease are strongest for the particulate components of air pollution. In the present study, to avoid the confounding influences of gaseous co-pollutants, DEP was administered to the lungs by intra-tracheal instillation, a highly reliable and reproducible method for producing excellent dispersion of particles throughout the lobes of rodent lungs and across the alveoli [17,24,25] This intervention resulted in increased blood pressure within 6 h of exposure. The dose of 0.5 mg, while high relative to doses achieved in inhalation studies, is nevertheless within the range used in other studies of particle exposure. Previous work in our laboratory has shown that while this dose of DEP increased blood pressure, it did not result in impaired in endothelial function or in systemic inflammation [17], suggesting that this instillation model does not result in acute severe toxicity, despite the relatively high level of exposure. Several neurohumoral mechanisms have been proposed to account for blood pressure increases in response to environmental pollution in experimental and clinical studies, including the release of endothelin, activation of the renin-angiotensin-aldosterone system and sympathetic activation [16,18]. Nonetheless, the mechanisms linking pulmonary exposure to pollutants with neurohumoral activation remain poorly defined. *In vitro* and *in vivo* studies have demonstrated that sensory receptors, including the transient receptor potential vanilloid type 1 (TRPV1), as well as TRPA1, can be activated by the cationic components of PM [20,21]. In the lung, activation of sensory receptors initiates a feedback response via the dorsal root, jugular and sometimes nodose ganglia to the CNS, with resultant effects on autonomic outflow [26]. In the present study, DEP associated elevation of systolic blood pressure was abrogated when the selective TRPV1 receptor antagonist, AMG 9810, was administered into the lung at the time of DEP instillation, supporting a key role for sensory receptor activation in mediating the hypertensive effects of DEP. Sympathetic nerves and TRP currents have previously been implicated in mediating cardiac electrophysiological instability associated with inhaled PM [10,11,20,21]. Findings here show that ischemia-associated arrhythmia was prolonged in rats that had been exposed to DEP and the severity of arrhythmia frequently led to death. This was also suppressed by pulmonary administration of the TRPV1 antagonist, lending further support to a key role for sensory nerve activation in mediating the effects of pulmonary DEP [10,11,20], and showing that activation of TRP is not dependent on the route of PM delivery. The data generated here hint at a link between sensory receptor and regulation of the sympathetic nervous system. However, further studies are required to fully understand the nature of this association through direct recording of sensory and sympathetic nerve fibre activation and of ion fluxes. Drugs that selectively block parasympathetic activation e.g. atropine, might also be included. This would reveal whether a true sensory reflex is evoked following exposure of the lung to particulate matter or whether other mechanisms, such as alteration of baroreflex sensitivity, are involved.

The incidence of fatal and non-fatal MI is increased clinically following exposure to air pollution [5], and this can occur within 6 h after exposure to fine particulate matter [27]. While some experimental studies have shown increased myocardial reperfusion injury 24 h after instillation of ultrafine particulate [13], or exposure to gaseous components of air pollution [28], the more acute effects and the underlying mechanisms remain largely unexplored. The primary aim of this study was to investigate whether susceptibility of the heart to ischemic injury was modified following acute exposure of the lung to diesel particulate and to gain some insight into mechanism. The study used a well-evaluated and characterised model of ischemia and reperfusion injury [17,29]. The data clearly shows that rats are rendered more vulnerable to *in vivo* myocardial ischemia and reperfusion within 6 h of DEP exposure with an increase in infarct size and an apparent increase in vascular permeability. In the intact animal, hemodynamic status or modification of neutrophil activation or recruitment to the injured myocardium could potentially underlie potentiation of myocardial injury. An increase in rate pressure product following instillation of DEP indicates that an increase in the energy demand of the heart, likely resulting from the increased blood pressure, might have contributed to increased injury associated with ischemia *in vivo*. Neutrophil numbers were also increased in blood after DEP exposure, although there was no concomitant increase in the concentration of the neutrophil chemoattractant molecules IL-8, or in IL-6 [30]. However, increased susceptibility to injury was reproduced *ex vivo* in hearts removed after *in vivo* DEP exposure, excluding an essential role for *in vivo* hemodynamic influences or blood borne inflammatory cells. This is supported by flow cytometric evaluation of granulocyte status that failed to reveal any evidence for neutrophil priming or activation following DEP exposure. These findings support the notion that modification of myocardial homeostasis following pulmonary exposure to components of air pollution might underlie the increased incidence of non-MI related cardiac death reported in epidemiological studies [7,8].

To address this issue further, hearts were investigated 6 h after exposure to DEP and prior to induction of MI. Increased oxidative stress generation was detected through direct measurement of reactive oxygen species in heart perfusate by EPR. This was associated with apoptosis and loss of cardiomyocyte viability. Local oxidant stress generation by the heart could be key in sensitizing the heart to injury, by inducing damage directly as shown, but also potentially by depleting the heart’s anti-oxidant capacity, leaving it more vulnerable to oxidant molecules generated during subsequent reperfusion [28]. Indeed, administration of antioxidant has previously been shown to prevent spontaneous myocardial arrhythmias induced by inhalation of concentrated ambient particles in rats [12]. Neutrophils are an important source of oxidant stress following reperfusion. It is very likely that oxidant stress associated with recruited neutrophils contributed to the apparent increase in vascular leak that followed *in vivo*, but not *ex vivo* I/R, in hearts from DEP exposed rats. However, histology confirmed that no neutrophils were present in hearts prior to induction of I/R. Neutrophils are not therefore likely to be responsible for the increase in baseline oxidant stress in hearts from DEP exposed rats. Other potential sources of the ROS in the myocardium include mitochondria, xanthine oxidase and nicotinamide adenine dinucleotide phosphate (NADPH) oxidase and several of these sources of free radicals are known to be upregulated following exposure to environmental particulates [30]. NADPH oxidases are of particular interest since these enzymes are widely distributed within the heart and they can be activated via β_1_ adrenoreceptor stimulation, either directly [31], or secondary to activation of the renin-angiotensin aldosterone system [32]. In the present study, increased baseline oxidant stress generation, apoptosis and loss of cardiomyocyte viability were prevented when metoprolol, a β_1_ adrenoreceptor antagonist with no direct anti-oxidant effects, was administered *in vivo* at the same time as DEP. Furthermore, this treatment also prevented sensitization of the isolated perfused heart to subsequent ischemia and reperfusion induced injury. As discussed above, autonomic activation following DEP exposure may occur secondary to activation of the pulmonary transient receptor potential cation (TRP) channels, resulting in increased blood pressure. TRP channel activation has also been implicated in mediating increased myocardial oxidant stress in rats exposed to inhaled diesel exhaust, or CAPs, although the specific components responsible have not been identified. [10,11]. To investigate their role in mediating apoptosis and sensitization to I/R in hearts from DEP exposed rats, the TRPV1 antagonist AMG 9810 was instilled into the lung *in vivo* with DEP 6 h prior to heart isolation. Blockade of TRPV1 completely prevented not only loss of cardiomyocyte viability prior to induction of ischemia but also the potentiation by DEP of reperfusion injury in isolated hearts. These observations support a key role for TRP activation in mediating the subsequent effects of intratracheal DEP at sites distant from the lung. While the TRP antagonist was administered into the pulmonary circulation, it is not possible to completely exclude blockade of receptors in the systemic circulation, that might also contribute to outcome. *Ex vivo* perfusion studies however suggest that a direct effect on cardiac TRPV1 does not account for the outcomes reported. *In vitro* experiments have shown that DEP interacts with TRPA1, in addition to TRPV1 [20,21]. Further experiments targeting blockade of these receptors are merited to further test their *in vivo* role.

The clinical relevance of these findings remains to be tested. There is growing clinical evidence that agrees with our observation of acute cardiovascular responses to particulate components of air pollution [2]. For example, changes in systolic blood pressure were found to occur within 30-60 minutes of acute exposure [23]. There are currently no controlled clinical studies of β adrenoreceptor antagonists or sensory neuron blockade in relation to pollution and cardiovascular outcomes. Interestingly though, de Hartog *et al.*[33] have demonstrated that associations between heart rate variability and exposure to traffic derived particulate matter are strongest in patients not taking β adrenoreceptor antagonists.

### Study limitations

(1) The study depends on the use of instillation for introduction of diesel particulate to the lungs and the dose selected, while non-toxic and similar to many other studies, is high relative to normal levels of exposure.

(2) While inhalation can also cause changes in blood pressure and electrical stability, there is a possibility that the responses reported here following instillation may not be triggered by real-world inhalation, and that humans may not respond in the same manner as the experimental animals used in this study.

## Conclusions

The data presented here demonstrate that within 6 h of a single exposure of the lung to DEP, by instillation, blood pressure is elevated and the myocardium is locally generating oxidant stress, resulting in injury, consistent with previous observations following inhalation of diesel exhaust [11,34-37]. Furthermore, the heart is rendered significantly more vulnerable to subsequent ischemic arrhythmia and to reperfusion-associated injury. While systemic inflammation and inflammatory cell recruitment appear not to be required, activation of sensory TRPV1 receptors, and of β_1_ adrenoreceptors are key to mediating these events.

## Materials and methods

### Animals

Adult male Wistar rats (200-250 g; Charles River, Margate, UK) were housed under controlled environmental conditions (21 ± 2°C; 12 h light/dark cycle) with access to tap water and standard laboratory rat chow *ad-libitum*. All rats were allowed to acclimatize to the environment for at least one week before experimental procedures were initiated. All experiments were performed according to the guidelines of the Animals (Scientific Procedures) Act 1986 (U.K. Home Office) and the National Institutes of Health (NIH Publication No. 85-23, revised 1996) and were approved by the ethical review committee for animal research at the University of Edinburgh.

### Intratracheal instillation of DEP

DEP (SRM-2975; National Institute of Standards and Technology, Gaithersburg, USA) was suspended in 0.9% sterile saline at a stock concentration of 1 mg/mL and sonicated for 5 min (70% power; 5 Hz) in an ice bath using a probe-type sonicator (US70; Philip Harris Scientific, Lichfield, U.K) to minimise particle aggregation.

DEP (0.5 mg) or an equivalent volume (0.5 mL) of 0.9% saline was administered by intra-tracheal instillation under light anaesthesia as previously described [38,39]. The dose used is comparable to previous studies [17,38,40,41]. An additional group of non-instilled rats was used to confirm that saline instillation itself did not contribute to myocardial injury.

### Ischemia/Reperfusion in vivo and ex vivo

6 h after instillation, rats were anesthetized (sodium pentobarbital (60 mg.kg^-1^ i.p.)) and prepared for hemodynamic assessment and for induction of ischemia and reperfusion (I/R) *in vivo*, or hearts were rapidly isolated and perfused in Langendorff mode *ex vivo* (for details, see Additional file 1: Methods). Infarct size was determined by triphenyltetrazolium chloride (TTC) staining (for details, see Additional file 1: Methods). In *ex vivo* perfused hearts, oxygen free radical generation was assessed in perfusate by spin-label electron paramagnetic resonance [29,42] using the spin trap 1-hydroxy-3-carboxy-pyrolidine (CP-H, 10^-3^ M) (for details, see Additional file 1: Methods).

### Drug treatment studies

Rats were randomly assigned to receive the β_1_-adrenoreceptor antagonist metoprolol (10 mg/kg i.p.)[43] or saline immediately before instillation, or the TRPV_1_ antagonist, AMG 9810 ((2E)-N-(2,3- Dihydro-1, 4-benzodioxin-6-yl)-3-[4-(1, 1- dimethylethyl)phenyl]-2-propenamide, 30 mg/kg) [44] intra-tracheally, dispersed in either DEP or in saline. In additional groups, *ex vivo* hearts underwent I/R while perfused with Tyrode’s containing metoprolol (10 μM) or AMG 9810 (1 μM), to control for any direct influence of the drugs on reperfusion injury.

### Drugs and reagents

Unless otherwise stated all pharmacological agents were obtained from Sigma Aldrich (Dorset, U.K.) and all basic salts were obtained from VWR (Leicestershire, U.K.). All drugs were dissolved in sterile 0.9% saline unless otherwise stated.

### Statistical analysis

Data are expressed as the mean ± standard error of the mean (SEM). Statistical comparisons were performed by one-way analysis of variance (ANOVA) followed by Bonferroni post-hoc tests (baseline hemodynamic, infarct size and EPR data) or two-way ANOVA using the Bonferroni post-hoc test (drug intervention studies), unless otherwise stated. Statistical analyses were performed using GraphPad Prism software (V5.0; GraphPad Software Inc, USA). Two-sided P < 0.05 was considered to be statistically significant.

## Abbreviations

AAR: Area at risk; ANOVA: Analysis of variance; CPP: Coronary perfusion pressure; DE: Diesel exhaust; DEP: Diesel exhaust particulate; EPR: Electron paramagnetic resonance; I/R: Ischemia and reperfusion; LV: Left ventricular; MI: Myocardial infarction; NADPH: Nicotinamide adenine dinucleotide phosphate; NIST: National Institute of Standards and Technology; PM: Particulate matter; RPP: Rate pressure product; SEM: Standard error of mean; TRPV1: Transient receptor potential vanilloid 1; TTC: Triphenyltetrazolium chloride.

## Competing interests

The authors declare that they have no competing interests.

## Authors’ contributions

SR participated in experimental design, carried out experimental work, performed the statistical analysis and drafted the manuscript. ALT, RC and HS contributed to experimental work and analysis. CAS, PWFH, DEN and MRM participated in the design of the study and/or helped to draft the manuscript. GAG conceived the study, participated in its design and coordination and drafted the manuscript. All authors read and approved the final manuscript.

## Supplementary Material

Additional file 1 Detailed methods, Figure S1, Tables S1-S4. Click here for file
