# Supplementary material for: Pulmonary diesel particulate increases susceptibility to myocardial ischemia/reperfusion injury via activation of sensory TRPV1 and β1 adrenoreceptors
Source: Part Fibre Toxicol. 2014 Feb 25;11:12. doi: 10.1186/1743-8977-11-12 (PMC4016506; doi:10.1186/1743-8977-11-12)
Supplement: Additional file 1 — Detailed methods, Figure S1, Tables S1-S4. [file 1743-8977-11-12-S1.doc]

**Additional file**

**Detailed Methods**

*Haemodynamic assessment and induction of ischemia/reperfusion in vivo* Six hours after instillation, all animals were anesthetised (sodium pentobarbital (60 mg.kg^-1^ i.p.)) and mechanically ventilated via the trachea (1.5 mL/100 g at 60 strokes/min). Polyethylene cannulae containing heparinised saline (100 IU/mL) were placed in the left carotid artery for blood pressure monitoring and into the right external jugular vein for drug infusion of additional anaesthetic as required. Limb lead electrocardiograms (ECG, right arm, left leg, and right leg leads) were monitored continuously using subcutaneous needle electrodes. The chest was opened via the ribs and myocardial ischemia was induced by placement of a ligature around the left anterior descending coronary artery. The ligature was released after 45 min and the heart was reperfused for a further 120 min, as previously described.[[6]](#_ENREF_6) The duration of periods of non-sinus rhythm (arrythmias, including tachycardia, ectopic beats and AV block) were recorded and added to give a total period of arrhythmia in different treatment groups. At the end of the reperfusion period, the left anterior descending coronary artery was re-occluded and Evans Blue (1% w/v in 1 mL saline) was injected into the jugular vein to differentiate the non-perfused area at risk (AAR) from the perfused myocardium. Hearts were rapidly removed, rinsed and frozen at -20°C until later analysis of infarct size, as described below.

*Ischemia/Reperfusion ex vivo* To assess whether hearts were primed *in vivo* for subsequent injury, studies were also carried out using hearts isolated from rats 6h after *in vivo* instillation. Hearts were rapidly removed from isofluorane-anesthetised rats and arrested in ice-cold buffer (modified Tyrode’s/HEPES buffer, pH 7.4, containing 140nM NaCl, 4 nM KCl, 1 nM MgCl_2_, 5 mM HEPES and 11.1 mM glucose). Hearts were then perfused at 37°C in a retrograde fashion via the aorta at a constant flow rate of 10 mL/min with oxygenated (95%O_2_/5%CO_2_) Tyrode’s buffer (containing 116 mM NaCl, 20 mM NaHCO_3_, 0.4 mM Na_2_HPO_4_, 1 mM MgSO_4_, 4 mM KCl, 11 mM glucose and 2.5 mM CaCl_2_). Coronary perfusion pressure (CPP) was measured continuously via the aortic cannula. Only hearts with perfusion pressure of 40-60 mmHg were used for the study. Following a 10 min equilibration, a ligature was placed around the left coronary artery for induction of ischemia. The ligature was loosened after 30 min and reperfusion was continued for a further 120 min. At this time the ligation was retied and Evans Blue (1% w/v in 1 mL saline) was injected into the aortic cannula to delineate the perfused and non-perfused (area at risk, AAR) tissue. Hearts were frozen at -20°C for later assessment of infarct size.

In *ex vivo* perfused hearts, oxygen free radical generation from the myocardium was assessed in perfusion fluid using spin-label electron paramagnetic resonance (EPR,[6, 7]): Coronary effluent was collected in 1 mL aliquots into tubes containing the spin trap 1-hydroxy-3-carboxy-pyrolidine (CPH-H, 10^-3^ M final concentration; Axxora Ltd, Nottingham, UK) and incubated at 37°C for 5 min. Pyrogallol was used as a positive control to generate superoxide radicals.[[8]](#_ENREF_8) Samples (50 µL) were measured with an X-band EPR spectrometer (Magnettech MS-200, Berlin, Germany) using the following settings: microwave power, 20 mW; modulation frequency, 100 kHz; modulation amplitude, 1500 mG; center field, 3365 G; sweep width, 43.95 G; sweep time, 30 sec; number of passes, 1). Spectra were quantified from the derivative of the peak-to-peak height of the EPR spectrum, expressed as arbitrary units (AU)

*Drug treatment studies* For interventional studies with β_1_-adrenoreceptor antagonism, rats were randomly assigned to receive either metoprolol (10 mg/kg i.p.) [9, 10] or saline vehicle immediately before instillation of DEP or saline. For the TRPV_1_ antagonist, rats were randomly assigned to receive AMG 9810 ((2E)-N-(2,3- Dihydro-1, 4-benzodioxin-6-yl)-3-[4-(1, 1- dimethylethyl)phenyl]-2-propenamide, 30 mg/kg) [[11]](#_ENREF_11) intra-tracheally dispersed in either DEP or in saline. In additional groups, hearts from DEP- or saline-instilled animals underwent *ex vivo* I/R while being perfused with Tyrode’s containing metoprolol (10 µM) [[12]](#_ENREF_12) or AMG 9810 (1 µM) [[13]](#_ENREF_13), to control for any direct *ex vivo* influence of the drugs on reperfusion injury.

*Measurement of infarct size by triphenyltetrazolium chloride (TTC) staining*

Myocardial infarct size was assessed by TTC staining as previously described.[[6]](#_ENREF_6) The investigator was blinded to the identity of the samples prior to assay. Briefly, frozen hearts were sliced into 2-3 mm thick transverse sections from apex to base and incubated in 1% TTC (w/v) at 37°C for 30 min. The slices were immersed in 10% formalin for 10 min to enhance the contrast between viable (TTC-stained red) and necrotic tissue (TTC-unstained) within the AAR. The infarcted area, the AAR, and the normally perfused region of the left ventricle were weighed. In some studies, in hearts from DEP-instilled rats, Evans Blue was unable to discriminate between perfused and non-perfused myocardium due to increased capillary leak. In these studies, infarct size was expressed as % left ventricular mass rather than % AAR.

*Whole blood cell count and assessment of neutrophil activation status by flow cytometry*

Heparin-anti-coagulated whole blood was withdrawn from the dorsal aorta of anaesthetised rats 6h after instillation of saline or DEP and immediately placed on ice. White blood cell (WBC), red blood cell (RBC) and platelet counts were measured with the Coutler®A^c^.T series analyser (Coutler Corporation, Miami, USA). Whole blood flow cytometry (FACS-Calibur, BD Pharmingen, Oxford, UK) was used to assess granulocyte activation by quantifying the expression of the cell surface molecular CD11b. Both non-stimulated and formyl-Met-Leu-Phe-OH (fMLP, 1 µg/mL)-stimulated samples were incubated at 37°C with gentle shaking for 30 min. Samples were placed on ice for 15 min and then incubated with a saturating concentration of the fluorescent-conjugated antibody against CD11b (mouse anti-rat mAb, clone OX-42, 10 µg/ml, AbD serotec, Oxford, UK) or the matched isotype control antibody (PE-conjugated mouse anti-rat IgG2a mAb, clone RP-1, BD Pharmingen, Oxford, UK). Following incubation in the dark for 20 min, erthrocytes were lysed with a 10X erytholyse buffer (1:10 dilution in distilled water, AbD Serotec, UK). After 10 min, cells were washed in PBS/ 1% BSA, centrifuged at 400 g for 5 min and re-suspended in PBS /1% BSA. Granulocytes were gated based on their distinct forward- and sideward-scatter profiles. Mean fluorescence intensity (MFI) of both CD11bLOW and CD11bHIGH expressing cells were recorded and analysis was performed using FlowJo Data Analysis Software.

“See Additional Information Methods & Additional file 1: Figure S1”

**Additional file 1: Figure S1 Pulmonary DEP instillation increased leukocyte count in the blood but was not associated with granulocyte priming.** White blood cell count (WBC, **a**) was significantly increased 6h after instillation of DEP (0.5mg), compared to saline (0.5ml) or no instillation, although there was no increased in the plasma concentration of the leukocyte mobilising chemokine CXCL8 (**b**). Flow cytometric evaluation of granulocytes collected at this time (**c**) did not demonstrate any basal priming (increase in CD11b ^hi^ cells), or any change in sensitivity to activation by formyl-Met-Leu-Phe-OH (fMLP, 1 µM). Results are expressed as mean ± SEM (n = 6 per group) *P<0.05 saline versus DEP; one-way ANOVA followed by Bonferroni post-hoc test

**Additional file 1: Table S1 Baseline values for heart rate, arterial blood pressure (systolic and diastolic) and rate pressure product after no instillation and 6 hours after instillation with saline or diesel exhaust particulate (DEP, 0.5 mg).**

|  | Group | | |
| --- | --- | --- | --- |
| Endpoint | Non-instilled | Saline | DEP |
| HR (beats/min) | 355 ± 12 | 358 ± 10 | 389 ± 13 |
| SBP (mmHg) | 113.0 ± 7.4 | 100.7 ± 7.8 | 148.5 ± 5.2*** |
| DBP (mmHg) | 101.8 ± 8.1 | 89.6 ± 5.7 | 139.3 ± 5.2*** |
| RPP (HR X SBP/1000) | 31.2 ± 3.1 | 32.4 ± 1.8 | 41.9 ± 2.5*** |

DEP, diesel exhaust particulate; HR, heart rate; SBP, systolic blood pressure; DBP, diastolic blood pressure; RPP, rate pressure product. Results are expressed as mean ± SEM (n=6). ***P<0.001, compared to saline, Bonferroni post-hoc test, following one-way ANOVA

**Additional file 1: Table S2 Haemodynamic parameters at time intervals immediately before induction of ischaemia, during ischaemia/reperfusion after no instillation and 6 hours after instillation with saline or diesel exhaust particulate (DEP – 0.5 mg).**

| Endpoint | Time | | | | |
| --- | --- | --- | --- | --- | --- |
|  | Prior to Isch | Onset Isch | 45 min Isch | 1 h Rep | 2 h Rep |
| ***Non-instilled*** |  | | | | |
| HR (beats/min) | 355 ± 12 | 341 ± 17 | 324 ± 14 | 330 ± 19 | 321 ± 20 |
| MAP (mmHg) | 105.5 ± 7.8 | 71.0 ± 2.6^##^ | 95.3 ± 4.0 | 90.7 ± 2.4 | 76.7 ± 2.6^##^ |
| RPP (HR X SBP/1000) | 31.3 ± 3.1 | 35.0 ± 1.0 | 30.5 ± 2.0 | 30.1 ± 1.6 | 24.7 ± 2.6 |
| ***Saline*** |  | | | | |
| HR (beats/min) | 358 ± 10 | 346 ± 10 | 335 ± 13 | 357 ± 7 | 342 ± 21 |
| MAP (mmHg) | 93.9 ± 6.4 | 59.4 ± 4.9^###^ | 96.8 ± 6.6 | 91.5± 4.1 | 68.9 ± 5.2 |
| RPP (HR X SBP/1000) | 32.4 ± 1.8 | 34.8 ± 2.0 | 34.0 ± 4.1 | 33.1 ± 1.8 | 28.6 ± 2.3 |
| ***DEP*** |  | | | | |
| HR (beats/min) | 389 ± 13 | 365 ± 12 | 366 ± 16 | 367 ± 10 | 347 ± 14 |
| MAP (mmHg) | 140.5 ± 6.2^***^ | 82.7 ± 10.7^###^ | 100.6 ± 8.5^###^ | 89.8± 2.4^###^ | 88.6 ± 2.8^###^ |
| RPP (HR X SBP/1000) | 41.9 ± 2.5^***^ | 46.6 ± 6.8 | 38.1 ± 5.1 | 38.3 ± 5.4 | 30.2 ± 2.9^#^ |

DEP, diesel exhaust particulate; Isch, ischaemia; Rep, reperfusion; HR, heart rate; MAP, mean arterial blood pressure; RPP, rate pressure product; SBP, systolic blood pressure

Results are expressed as mean ± SEM (n=4-6). ***P<0.001 versus saline; #P<0.05, ##P<0.01, ###P<0.001 versus baseline in each group; two-way ANOVA with Bonferroni post-hoc test.

**Additional file 1: Table S3 Baseline values for coronary perfusion pressure (CPP) and area at risk (AAR) in control hearts and in hearts isolated from rats previously treated *in vivo* with AMG 9810 or metoprolol at the time of saline or DEP instillation.**

| ***In vivo***  **drug treatment** | | **CPP (mmHg)** | | | **AAR (%LV)** | | |
| --- | --- | --- | --- | --- | --- | --- | --- |
|  |  | *In vivo* instillation | | | *In vivo* instillation | | |
|  |  | *non-instilled* | *saline* | *DEP* | *non-instilled* | *saline* | *DEP* |
| **control** | - | 45.0 ± 6.1 | 48.8 ± 4.1 | 52.5 ± 3.0 | 3.3 ± 3.3 | 81.3 ± 2.0 | 76.8 ± 3.0 |
| **AMG 9810 (i.t.)** | saline | - | 49.1 ± 9.3 | 41.6 ± 5.7 | - | 75.4 ± 6.2 | 77.2 ± 3.2 |
|  | AMG 30 mg/kg | - | 39.7 ± 3.9 | 55.4 ± 2.2 | - | 74.1 ± 5.6 | 74.8 ± 4.5 |
| **Metoprolol (i.p.)** | saline | - | 52.7 ± 5.4 | 52.5 ± 3.0 | - | 81.3 ± 2.0 | 76.8 ± 3.0 |
|  | Metoprolol 10 mg/kg | - | 51.8 ± 6.5 | 59.8 ± 2.6 | - | 79.2 ± 2.3 | 76.0 ± 4.0 |

no instillation; saline, 6h after instillation of saline; DEP, 6 h after instillation of diesel exhaust particulate, 0.5mg; LV, left ventricle, i.t. intratracheal, i.p.intraperitoneal; Results are expressed as mean ± SEM (n=4-6). No significant differences were observed within any of the studies.

**Additional file 1: Table S4. Baseline values for coronary perfusion pressure (CPP) and area at risk (AAR) in hearts isolated from rats treated *in vivo* with vehicle or DEP and subsequently exposed to AMG 9810 or metoprolol *ex vivo* in buffer perfusate.**

| **Drug & Perfusate Concentration** | **CPP (mmHg)** | | | **AAR (%LV)** | | |
| --- | --- | --- | --- | --- | --- | --- |
|  | *In vivo* instillation | | | *In vivo* instillation | | |
|  | *non-instilled* | *saline* | *DEP* | *non-instilled* | *saline* | *DEP* |
| **none** | 45.0 ± 6.1 | 48.8 ± 4.1 | 52.5 ± 3.0 | 73.3 ± 3.3 | 81.3 ± 2.0 | 76.8 ± 3.0 |
| **AMG 9810**  **(1 µM)** | - | 45.4 ± 9.0 | 45.2 ± 8.2 | - | 69.8 ± 5.9 | 62.4 ± 18.9 |
| **Metoprolol**  **(10µM)** | - | - | 60.5 ± 3.4 | - | - | 83.1 ± 2.3 |

non-instilled: no instillation; saline, 6h after instillation of saline; DEP, 6 h after instillation of diesel exhaust particulate, 0.5mg; LV, left ventricle. Results are expressed as mean ± SEM (n=4-6). No significant differences were observed within any of the studies.

**References**

1. McQueen DS, Donaldson K, Bond SM, McNeilly JD, Newman S, Barton NJ, Duffin R: **Bilateral vagotomy or atropine pre-treatment reduces experimental diesel-soot induced lung inflammation.** *Toxicol Appl Pharmacol* 2007, **219:**62-71.

2. Madden MC, Richards JH, Dailey LA, Hatch GE, Ghio AJ: **Effect of ozone on diesel exhaust particle toxicity in rat lung.** *Toxicol Appl Pharmacol* 2000, **168:**140-148.

3. Yokota S, Seki T, Furuya M, Ohara N: **Acute functional enhancement of circulatory neutrophils after intratracheal instillation with diesel exhaust particles in rats.** *Inhal Toxicol* 2005, **17:**671-679.

4. Murphy SA, BeruBe KA, Pooley FD, Richards RJ: **The response of lung epithelium to well characterised fine particles.** *Life Sci* 1998, **62:**1789-1799.

5. Yan YH, Huang CH, Chen WJ, Wu MF, Cheng TJ: **Effects of diesel exhaust particles on left ventricular function in isoproterenol-induced myocardial injury and healthy rats.** *Inhal Toxicol* 2008, **20:**199-203.

6. Jeanes HL, Tabor C, Black D, Ederveen A, Gray GA: **Oestrogen-mediated cardioprotection following ischaemia and reperfusion is mimicked by an oestrogen receptor (ER)alpha agonist and unaffected by an ER beta antagonist.** *J Endocrinol* 2008, **197:**493-501.

7. Miller MR, Borthwick SJ, Shaw CA, McLean SG, McClure D, Mills NL, Duffin R, Donaldson K, Megson IL, Hadoke PW, Newby DE: **Direct impairment of vascular function by diesel exhaust particulate through reduced bioavailability of endothelium-derived nitric oxide induced by superoxide free radicals.** *Environ Health Perspect* 2009, **117:**611-616.

8. Taylor EL, Rossi AG, Shaw CA, Dal Rio FP, Haslett C, Megson IL: **GEA 3162 decomposes to co-generate nitric oxide and superoxide and induces apoptosis in human neutrophils via a peroxynitrite-dependent mechanism.** *Br J Pharmacol* 2004, **143:**179-185.

9. Beique JC, Blier P, de Montigny C, Debonnel G: **Potentiation by (-)Pindolol of the activation of postsynaptic 5-HT(1A) receptors induced by venlafaxine.** *Neuropsychopharmacology* 2000, **23:**294-306.

10. Beril Gok H, Solaroglu I, Okutan O, Cimen B, Kaptanoglu E, Palaoglu S: **Metoprolol treatment decreases tissue myeloperoxidase activity after spinal cord injury in rats.** *J Clin Neurosci* 2007, **14:**138-142.

11. Gavva NR, Tamir R, Qu Y, Klionsky L, Zhang TJ, Immke D, Wang J, Zhu D, Vanderah TW, Porreca F, et al: **AMG 9810 [(E)-3-(4-t-butylphenyl)-N-(2,3-dihydrobenzo[b][1,4] dioxin-6-yl)acrylamide], a novel vanilloid receptor 1 (TRPV1) antagonist with antihyperalgesic properties.** *J Pharmacol Exp Ther* 2005, **313:**474-484.

12. Kovacs K, Hanto K, Bognar Z, Tapodi A, Bognar E, Kiss GN, Szabo A, Rappai G, Kiss T, Sumegi B, Gallyas F, Jr.: **Prevalent role of Akt and ERK activation in cardioprotective effect of Ca(2+) channel- and beta-adrenergic receptor blockers.** *Mol Cell Biochem* 2009, **321:**155-164.

13. Henrich M, Buckler KJ: **Acid-evoked Ca2+ signalling in rat sensory neurones: effects of anoxia and aglycaemia.** *Pflugers Arch* 2009, **459:**159-181.
